# Dietary Shifts May Trigger Dysbiosis and Mucous Stools in Giant Pandas (*Ailuropoda melanoleuca*)

**DOI:** 10.3389/fmicb.2016.00661

**Published:** 2016-05-06

**Authors:** Candace L. Williams, Kimberly A. Dill-McFarland, Michael W. Vandewege, Darrell L. Sparks, Scott T. Willard, Andrew J. Kouba, Garret Suen, Ashli E. Brown

**Affiliations:** ^1^Department of Biochemistry, Molecular Biology, Entomology and Plant Pathology, Mississippi State UniversityMississippi State, Mississippi, MS, USA; ^2^Institute for Genomics, Biocomputing and Biotechnology, Mississippi State UniversityMississippi State, Mississippi, MS, USA; ^3^Department of Bacteriology, University of Wisconsin-MadisonMadison, WI, USA; ^4^Mississippi State Chemical LaboratoryMississippi State, Mississippi, MS, USA; ^5^Department of Conservation and Research, Memphis Zoological SocietyMemphis, TN, USA

**Keywords:** 16S rRNA sequencing, mucoid, fecal microbiota, mucosal microbiota, bamboo part preference

## Abstract

Dietary shifts can result in changes to the gastrointestinal tract (GIT) microbiota, leading to negative outcomes for the host, including inflammation. Giant pandas (*Ailuropoda melanoleuca*) are physiologically classified as carnivores; however, they consume an herbivorous diet with dramatic seasonal dietary shifts and episodes of chronic GIT distress with symptoms including abdominal pain, loss of appetite and the excretion of mucous stools (mucoids). These episodes adversely affect the overall nutritional and health status of giant pandas. Here, we examined the fecal microbiota of two giant pandas’ non-mucoid and mucoid stools and compared these to samples from a previous winter season that had historically few mucoid episodes. To identify the microbiota present, we isolated and sequenced the 16S rRNA using next-generation sequencing. Mucoids occurred following a seasonal feeding switch from predominately bamboo culm (stalk) to leaves. All fecal samples displayed low diversity and were dominated by bacteria in the phyla Firmicutes and to a lesser extent, Proteobacteria. Fecal samples immediately prior to mucoid episodes had lower microbial diversity as compared to mucoids. Mucoids were mostly comprised of common mucosal-associated taxa including *Streptococcus* and *Leuconostoc* species, and exhibited increased abundance for bacteria in the family Pasteurellaceae. Taken together, these findings indicate that mucoids may represent an expulsion of the mucosal lining that is driven by changes in diet. We suggest that these occurrences serve to reset their GIT microbiota following changes in bamboo part preference, as giant pandas have retained a carnivorous GIT anatomy while shifting to an herbivorous diet.

## Introduction

The host-symbiont relationship within the gastrointestinal tract (GIT) of animals is critical, as these symbionts play a fundamental role in fiber digestion, modulation of the host immune system, and maintenance of host-bacterial homeostasis (Hooper et al., 2002; Flint et al., 2012). In particular, microorganisms associate with the gastrointestinal lymphoid tissue to exclude pathogens and produce short chain fatty acids (SCFAs) that serve as an energy source for the host (Johansson et al., 2011; Flint et al., 2012). Also, SCFAs cause the intestinal epithelial cells (IEC) to increase expression of tight junction proteins, thus further increasing the barrier to pathogens (Brown et al., 2003; Louis and Flint, 2009).

The GIT biology of giant pandas (*Ailuropoda melanoleuca*) is peculiar because they are evolutionarily related to carnivores and possess the GIT morphology of a carnivore, yet they consume an exclusively herbivorous diet. This feature is surprising, given that the switch from an omnivorous to an herbivorous diet occurred approximately 2 to 2.4 million years ago (Davis, 1964; Schaller et al., 1985; Jin et al., 2007), yet giant pandas have not evolved adaptations seen in traditional herbivores, like a rumen or an enlarged cecum, to aid in fiber degradation. It remains unclear how pandas persist solely on bamboo, as they consume large amounts of the fibrous plant (Schaller et al., 1985), relative to other herbivores of their size. However, it has been suggested that they rely on bamboo’s hemicellulose content, rather than more difficult to digest cell wall components such as lignin and cellulose (Dierenfeld et al., 1982).

Both wild and captive pandas annually undergo dramatic shifts in bamboo part preference between culm (stalk) and leaves (Schaller et al., 1985; Tarou et al., 2005; Hansen et al., 2010; Williams et al., 2012) resulting in significant changes in fecal consistency and GIT microbial communities (Nickley, 2001; Williams et al., 2012; Xue et al., 2015). These dietary shifts have been attributed to changes in bamboo composition, as Schaller et al. (1985) found levels of silica to increase in the leaf portion of bamboo during times when pandas preferred the culm portion. This increase in silica content has been associated with anti-herbivory defense pathways in plants, which may explain why pandas undergo such a dramatic change in diet preference (Schaller et al., 1985; Ito and Sakai, 2009).

These endangered bears also suffer greatly from GIT disorders both *ex situ* and *in situ* (Qiu and Mainka, 1993; Loeﬄer et al., 2006). In humans, when the host-gut microbe relationship is severely disturbed, a condition termed dysbiosis can occur, and the host can experience an inflammatory response; if unchecked, this can develop into a chronic condition (Fava and Danese, 2011). Similarly, captive giant pandas undergo chronic GIT distress, with bouts of abdominal discomfort and loss of appetite, resulting in the excretion of a mucous-like stool (mucoid), although no investigation into their composition has occurred to date (Edwards et al., 2006; Loeﬄer et al., 2006). Necropsies from pandas that chronically suffer from this condition often show evidence of ulcerative and necrotizing suppurative colitis (Loeﬄer et al., 2006).

While mucoid occurrence has been associated with the presence of some pathogenic microorganisms (Loeﬄer et al., 2006), a direct link between specific pathogens and mucoids has not been found. Increases in dietary protein are known to result in greater occurrences of mucoids (Edwards et al., 2006; Janssen et al., 2006), suggesting that diet may be the underlying cause. However, captive giant pandas fed a high-fiber bamboo diet still commonly experience mucoids, so the cause and means to prevent these episodes remains unclear. The timing of mucoids is also critical, as they typically occur during a seasonal dietary shift directly following the breeding season, and any decreased nutritional status during gestation or lactation may affect offspring (Steinman et al., 2006; Zhang et al., 2006). Here, we used next-generation sequencing to characterize the fecal- and mucoid-associated microbiota in two giant pandas and to determine if drastic changes in diet correlate to a concomitant shift in the GIT microbiota and the expulsion of mucoids. Comparison of the bacterial communities associated with mucoid episodes (mucoid) to fecal samples, both within (non-mucoid) and outside the sample season (winter), provides insights into possible microbial contributions to this important chronic ailment in giant pandas.

## Materials and Methods

### Study Animals

The two giant pandas (“YaYa,” female, studbook number: 507, and “LeLe,” male, studbook number: 466) used in this study were housed at the Memphis Zoological Society, Memphis, TN, USA. Samples were collected under a signed biomaterials request form, and no IACUC protocol was needed as this project was viewed as non-invasive by the institution.

### Behavior Analysis of Bamboo Consumption

The study of bamboo consumption behavior at the Memphis Zoo has been ongoing since the fall of 2003 and was conducted as previously described (Hansen et al., 2010; Williams et al., 2012). In brief, behavior data were collected in 20-min periods in 30-s increments while the bear was feeding on bamboo using an ethogram focusing on foraging behaviors. These behaviors were divided into three consumption categories: leaf, culm (stalk), and other (shoot or branch). For each month, the total consumption behaviors were quantified by time spent consuming specific parts and each individual’s behavior was expressed as a percentage of the total consumption behaviors.

### Sample Collection

Fresh fecal (*n* = 5 female, 13 male) and mucous excretion (*n* = 1, 5) samples were collected. All samples were transported on dry ice, and stored at –80°C prior to processing. Samples were classified as “winter,” “non-mucoid,” or “mucoid” (Supplementary Table S1). Winter control samples (*n* = 5,5) were collected on 02/12/13, during a season with historically low mucoid occurrence and prior to first mucoid excretion in this study. Additional sample collection occurred between 6/29/14 and 8/22/14. During this period, collected male and female stool samples were categorized into non-mucoid or mucoid movements. The date of the movement was also recorded to study temporal changes. Of note, the male produced a mucoid sample on 07/17 (day 14) that was not successfully sequenced but a non-mucoid fecal sample on this date was.

### DNA Extraction

Total genomic DNA from fecal samples was extracted via mechanical disruption and hot/cold phenol extraction following the protocol described by Stevenson and Weimer (2007) with the following modification: 25:24:1 phenol:chloroform:isoamyl alcohol was used in place of phenol:chloroform at all steps. DNA was quantified using a Qubit Fluorometer (Invitrogen) and stored at –20°C following extraction.

### Library Preparation and Sequencing

Library preparation was carried out following manufacturer’s recommendations (Illumina, 2013) with some modifications. In brief, an amplicon PCR targeted the V3–V4 region of the 16S rRNA gene using a forward (V3-4F, TCGTCGGCAGCGTCAGATGT GTATAAGAGACAGCCTACGGGNGGCWGCAG) and reverse (V3-4R, GTCTCGTGGGCTCGGAGATGTGTATAAGAGACAGGC TACHVGGGTATCTAATCC) primer (Klindworth et al., 2013) in a 25-μL reaction with 1X KAPA HiFi Hot Start Ready Mix (Kapa Biosystems), 0.2 mM each primer, and 1–10 ng DNA. Amplification conditions were as follows: 95°C for 3 min, 25 cycles of 95°C for 30 s, 55°C for 30 s, 72°C for 30 s, and a final elongation of 72°C for 5 min. PCR products were purified via gel extraction (Zymo Gel DNA Recovery Kit; Zymo, Irvine, CA, USA, USA) using a 1% low melt agarose gel (National Diagnostics, Atlanta, GA, USA). Purified products underwent an indexing 25 μL-PCR reaction (1x KAPA HiFi Hot Start Ready Mix, 0.2 mM indices, and 5 μL of purified product) with the same reaction conditions as amplicon PCR with the exception of a reduction in the number of cycles to 8.

The final index PCR product underwent gel extraction (Zymo Gel DNA Recovery Kit; Zymo, Irvine, CA, USA), and the resulting purified product concentration was determined by a Qubit Fluorometer (Invitrogen). Samples were combined to yield an equimolar 4 nM pool. Following manufacturer’s protocol, sequencing was conducted on an Illumina MiSeq using reagent kit V3 (2 x 300 bp cycles), as described previously (Illumina, 2013). All sequences were deposited into the National Center for Biotechnological Information’s Short Read Archive under Accession Number SRP065974.

### Data Analysis

Sequence analysis was carried out using mothur v.1.34.1 following the MiSeq SOP (Kozich et al., 2013). In brief, contigs were formed from 16S rRNA reads, and poor quality sequences were removed. Sequences were trimmed and filtered based on quality (maxambig = 0, minlength = 250, maxlength = 600). Unique sequences were aligned against the SILVA 16S rRNA gene alignment database (Pruesse et al., 2007) and classified with a bootstrap value cutoff of 80, and operational taxonomic units (OTUs) found with <2 sequences in the total dataset were removed. Chimeras (chimera.uchime) and sequences identified as members of Eukaryota, Archaea, and Cyanobacteria lineages were also removed.

### Statistical Analyses

Sequence coverage was assessed in mothur by rarefaction curves and Good’s coverage (Good, 1953). Samples were then iteratively subsampled 10 times to 600 sequences per sample, and OTU abundances were calculated as the whole-number means across iterations. Differences in bacterial community were visualized by non-metric dimensional scaling plots (nMDS, iters = 10,000; Shepard, 1966) of Bray-Curtis (Bray and Curtis, 1957) and Jaccard (Jaccard, 1912) similarity (beta-diversity) indices, also calculated in mothur.

All other statistical analyses were carried out in R [vegan package (Oksanen et al., 2015; R Core Team, 2015)] or SAS 9.3 software (Cary, NC, USA), and data were expressed as the mean ± SEM and considered significant if *P* < 0.05. In R, differences in taxonomic profiles were assessed at the phyla, family, and OTU levels. Due to uneven sampling, analysis of similarity (ANOSIM) was used to compare community structure (Bray–Curtis; Bray and Curtis, 1957) and community composition (Jaccard; Jaccard, 1912) of winter, non-mucoid, and mucoid sample types. Samples were randomized with respect to sample type and tested to ensure true significance. Similarity percentages (SIMPER) analyses were then used to determine the contributions of taxonomic groups to differences observed in the ANOSIM. In SAS, the general linearized model (PROC GLM) was used to determine if diversity differed with respect to sample type.

## Results

### Bamboo Consumption Behavior

**FIGURE 1:**
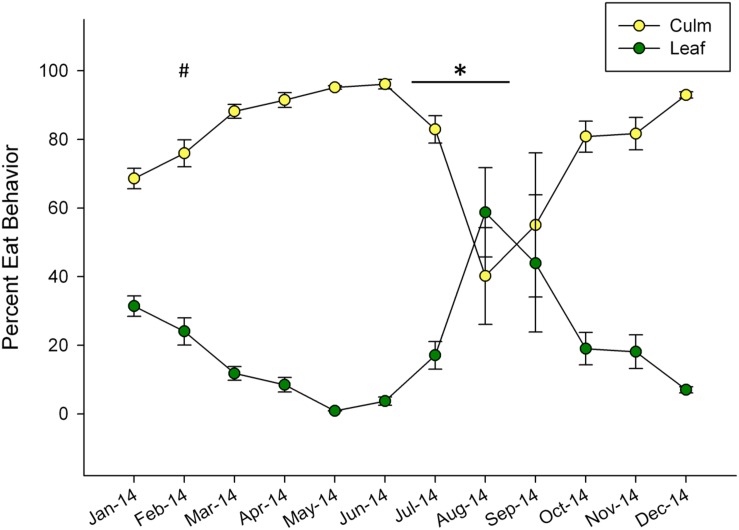
**Mean ± SEM monthly proportion of bamboo consumption behavior observed for leaf and culm displayed as percentage of total feeding observations for two giant pandas over a single year.** Winter (#) and non-mucoid/mucoid (^∗^) fecal sampling timeframes are indicated.

Dramatic shifts in eating behavior were observed in both pandas (**Figure 1**). In general, the bears consumed more culm than leaf throughout the year, but shifted to higher proportions of leaf consumption for the months of August and September. The pandas consumed negligible amounts of leaf material in May (0.88%) and increased their leaf consumption to its highest relative proportion in August, (59%) around the time of mucoid sampling in this study. Following this peak, leaf consumption steadily declined through December (**Figure 1**).

### Sequence Coverage and Taxonomy

For all samples (*n* = 34), a total of 457,358 raw and 375,406 high-quality (11,376 ± 2,170 sequences per sample) 16S rRNA sequences were generated using Illumina MiSeq paired-end sequencing (Supplementary Table S2). A Good’s coverage value of >0.99 (Supplementary Table S2) and a leveling off of rarefaction curves (Supplementary Figure S1) indicated that sequencing was adequate to detect the majority of bacterial diversity present in all samples. A 97% OTU analysis corresponding to species-level classification (Schloss and Handelsman, 2005) identified 118 unique OTUs across all samples with 14 to 84 OTUs per sample type (Supplementary Table S2).

Sequences from 15 phyla were found across all samples, with 70 ± 5.8% belonging to the Firmicutes and 28 ± 5.7% belonging to the Proteobacteria (Supplementary Figure S2). All other phyla represented less than 1.0% relative sequence abundance. Bacterial classes with >1.0% included the Clostridia (40 ± 4.9%), Gammaproteobacteria (27 ± 5.7%), Erysipelotrichia (16 ± 2.9%), and Bacilli (15 ± 3.2, Supplementary Figure S2). Orders with >1.0% representation corresponded to the Clostridiales (30 ± 4.9%), Enterobacteriales (25 ± 5.7%), Erysipelotrichales (16 ± 2.9%), Lactobacillales (15 ± 3.2%), and Pasteurellales (1.3 ± 0.65%, Supplementary Figure S2). At the family and genus levels, 99 and 98% of the sequences were annotated, respectively.

### Sample Type Affects Overall Bacterial Diversity

**FIGURE 2:**
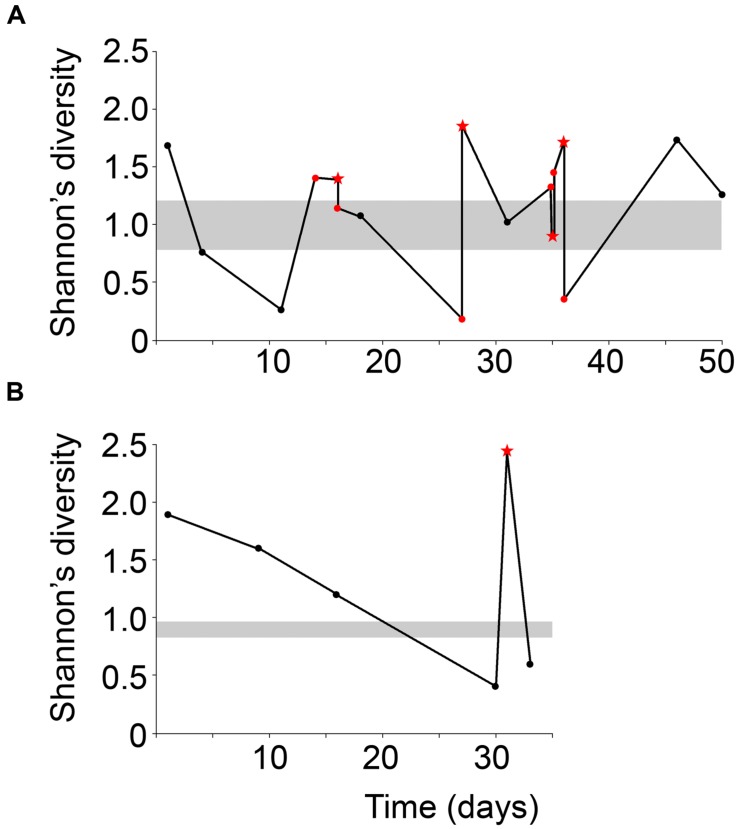
**Shannon’s diversity index for the (A) male and (B) female giant panda over the sampling period.** Non-mucoid feces are black dots and non-mucoid feces occurring on the same day as a mucoid are represented by red dots. Mucoids are red stars. The ranges of winter values are shaded in gray. The male panda experienced a mucoid on day 14 but this sample was not successfully sequenced and is not included here.

Mucous stools (mucoid) and fecal samples (non-mucoid) were obtained from the same season and compared to fecal samples from a historically low-mucoid season (winter). Sample diversity varied over the sampling period for both male and female giant pandas (**Figure 2**). In particular, both male and female displayed higher Shannon’s diversity than winter samples. At the beginning of mucoid season sampling diversity decreased dramatically prior to the appearance of the first mucoid (**Figure 2**). Overall, mucoid samples from both pandas displayed higher diversity, as measured by indices taking into account both presence and abundance of all taxa in the sample (Shannon: 1.7 ± 0.26, inverse-Simpson: 4.0 ± 1.0), than winter and non-mucoid fecal samples (Shannon, ANOVA, *P* = 0.0166). Although not significant, these samples were less dominated by single OTUs (inverse Berger-Parker: 2.6 ± 0.56, *P* > 0.05, **Table 1**). Non-mucoid fecal samples displayed the lowest average diversity with the highest variation (Shannon: 1.1 ± 0.13, inverse-Simpson: 2.6 ± 0.32) of all sample types, and were dominated by a single OTU (inverse Berger-Parker: 1.9 ± 0.20; Supplementary Table S2).

**Table 1:** Percent relative abundance (RA) and percentage contribution to defining differences between sample types at different taxonomic levels, as determined by SIMPER analysis, for panda winter, non-mucoid, and mucoid samples.

Comparison	Phyla	RA (%)	SIMPER (%)	Family	RA (%)	SIMPER (%)	OTU	Lowest classification	RA (%)	SIMPER (%)
Winter: Non-mucoid	Firmicutes	98:57	49	Clostridiaceae	57:23	28	1	*Clostridium*	57:23	28
				Erysipelotrichaceae	31:8.4	18	3	*Turicibacter*	31:8.4	18
	Proteobacteria	1.2:43	50	Enterobacteriaceae	0.22:43	31	2	*Escherichia-Shigella*	0.22:43	31
Non-mucoid: Mucoid	Firmicutes	57:64	46	Clostridiaceae	23:35	23	1	*Clostridium*	23:35	22
				Erysipelotrichacae	8.4:12	9.4	3	*Turicibacter*	8.4:12	9.0
				*Leuconostoc*acae	13:0.0	9.5	6	*Leuconostoc*	11:0.0	7.5
				Streptococcaceae	8.1:8.2	8.0	4	*Streptococcus*	7.4:0.63	5.3
							12	*Streptococcus*	0.0:7.2	5.1
	Proteobacteria	43:29	45	Enterobacteriaceae	43:13	27	2	*Escherichia-Shigella*	43:13	26
Winter: Mucoid	Firmicutes	98: 64	49	Clostridiaceae	57:35	29	1	*Clostridium*	57:35	28
				Erysipelotrichaceae	31:12	22	3	*Turicibacter*	31:12	21
				Streptococcaceae	5.7:8.2	9.1	12	*Streptococcus*	0.0:7.2	6.3
	Proteobacteria	1.2:29	38	Enterobacteriaceae	0.22:13	12	2	*Escherichia-Shigella*	0.22:13	11
							11	Family Pasteurellaceae	0.0:6.1	5.3

*RA, Relative abundance.*

### Overall Fecal Communities Differ According to Sample Type

**FIGURE 3:**
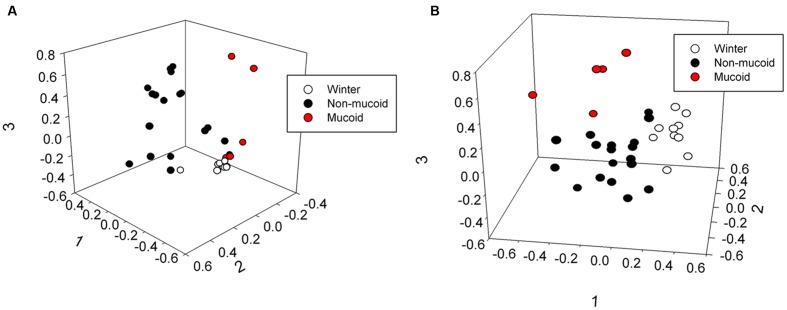
**Three-dimensional non-metric multidimensional scaling analysis showing differences in (A) community structure (Bray–Curtis, lowest stress: 0.0810, R-square: 0.965) and (B) community composition (Jaccard, lowest stress: 0.192, R-square: 0.766) of winter, non-mucoid, and mucoid samples in giant pandas**.

The male and female samples were grouped by sample type (winter, non-mucoid, and mucoid), and total bacterial community structure (Bray–Curtis) and composition (Jaccard) within the winter and non-mucoid groups, with sample types tested both individually and combined, did not significantly differ by animal (ANOSIM, *P* > 0.05, Supplementary Tables S3 and S5). The mucoid group could not be tested as the female had only one mucoid sample. Fecal communities were found to differ by sample type, as statistical analysis revealed differences in community structure (Bray–Curtis) at the phyla (ANOSIM, *P* = 0.035), family (*P* = 0.00030) and OTU levels (*P* = 0.00040) (Supplementary Table S4). Community composition (Jaccard) was also found to vary significantly with respect to sample type across all three taxonomic levels (ANOSIM, *P* = 0.040, 0.0007, and 0.00040, respectively; Supplementary Table S4). These differences in overall bacterial community composition and structure were visualized by non-metric dimensional scaling (nMDS; **Figure 3**; Supplementary Figure S3). No significant differences with respect to sample type were observed when randomized (ANOSIM, *P* > 0.05; Supplementary Table S4).

### Few Taxonomic Groups Shape Overall Bacterial Community

To determine which taxonomic groups contributed to the significant differences observed between sample types, analyses at the phyla, family and OTU levels were conducted. Only two phyla, the Proteobacteria and the Firmicutes, were found to drive differences between the three sample types [SIMPER, contribution to overall dissimilarity: winter-non-mucoid comparison (WN): 50 and 49%, respectively; winter-mucoid comparison (WM): 40 and 49%, respectively; non-mucoid-mucoid comparison (NM): 45 and 46%)] (**Table 1**). Family members of these phyla also contributed to the differences observed, with five families found to be important in sample comparisons. For WN, the Enterobacteriaceae, Clostridiaceae, and Erysipelotrichaceae, were found to be important drivers (SIMPER, contribution to overall dissimilarity: 31, 28, and 18%, respectively; **Table 1**). These three families, with the addition of the Streptococcaceae, were found to significantly shape differences in WM comparisons (**Table 1**). An additional family, the Leuconostocaceae, was also observed to significantly drive differences in NM bacterial communities (**Table 1**).

**FIGURE 4:**
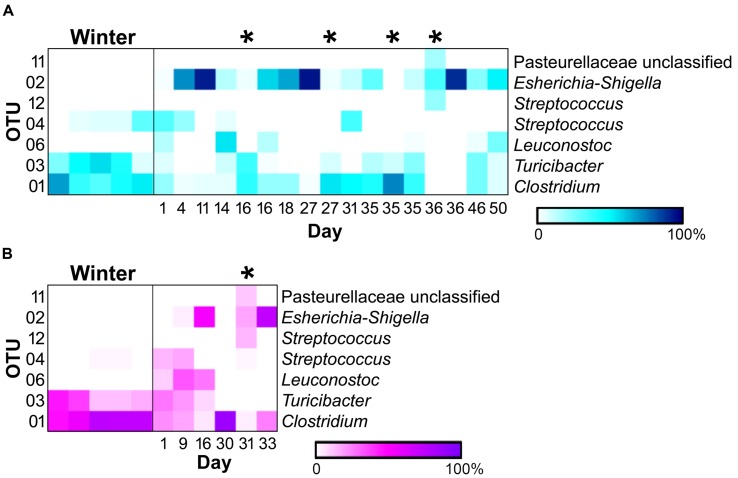
**Heatmaps of relative abundances of operational taxonomic units (OTUs) significantly contributing to differences seen between sample types (SIMPER) in the (A) male (winter = 5, non-mucoid = 13, mucoid = 4) and (B) female giant panda (5, 5,and 1).** OTU classifications are indicated to the right, and mucoids are marked by (^∗^).

Of the 118 OTUs observed in the samples, only six were found to significantly contribute to the differences seen in the sample types (**Figure 4**). Three OTUs, an *Escherichia-Shigella* species (OTU 2), a *Clostridium* species (OTU 1), and a *Turicibacter* species (OTU 3), were influential in shaping differences in the WN comparison (SIMPER, contribution to overall dissimilarity: 31, 28, and 18%, respectively; **Table 1**). These, as well as an additional two OTUs, contributed to differences observed in BM: a *Streptococcus* species (OTU 12) and an unclassified member of the Pasteurellaceae (OTU 11; **Table 1**). For the NM analysis, all of the previously observed OTUs, except OTU 11, contributed to differences as well as a *Streptococcus* species (OTU 4) and a *Leuconostoc* species (OTU 6) (**Table 1**).

## Discussion

Here, we characterized the bacterial microbiota associated with fecal and mucous stools in giant pandas and correlated these communities to feeding shifts to determine the dietary and microbial contributions to mucoid episodes, a chronic and detrimental condition among these herbivorous carnivores. Fecal samples in this study were grouped as feces from a non-mucoid season (winter), feces immediately preceding or following a mucoid episode (non-mucoid), or mucus stools (mucoid).

Consistent with previous reports (Zhu et al., 2011; Xue et al., 2015), all fecal samples had low diversity (**Table 1**) and were dominated by bacteria in the phyla Firmicutes and Proteobacteria, with substantial contributions from the genera *Clostridium*, *Escherichia-Shigella*, *Streptococcus* and *Turicibacter*. Mucoid season samples (non-mucoid and mucoid) were obtained from a predominately leaf-eating season and tended to have less abundant *Clostridium* species and more abundant members of the family Enterobacteriaceae, particularly *Escherichia-Shigella*. Similar seasonal trends were observed previously with the same animals using culture techniques (Williams et al., 2012). However, the opposite trends were previously reported using next-generation sequencing of samples across a dietary change to more leaf for both wild and captive giant pandas (Xue et al., 2015). This discrepancy could be due to dietary differences as the pandas in the Xue et al. (2015) study consumed a different species of bamboo, as well as steamed bread, throughout the study. Importantly, it was not noted that consumption of bread varied with bamboo portion preference. Also, a wide range of methodological differences between the two studies, such as fecal sample processing (blending vs. vortexing), DNA extraction, or primer bias, may account for the observed differences (Brooks et al., 2015; Wagner Mackenzie et al., 2015).

When examining phyla-level contributions to sample type differences, there is a shift in the Gram-positive to Gram-negative ratio due to changes in the relative abundances of the phyla Firmicutes and Proteobacteria. The Firmicutes (Gram-positive) are commonly considered protective commensals (Craven et al., 2012) and dominated winter samples, but were less abundant in non-mucoid and mucoid samples. Inversely, the Proteobacteria (Gram-negative) include aggressive, pathogenic species (Baumgart et al., 2007; Craven et al., 2012), and they were found to be increased in the non-mucoid and mucoid samples, relative to winter. The largest changes in both phyla were observed between the winter and non-mucoid samples, indicating that these giant pandas experiences significant changes within their microbiota between seasons, possibly as a result of differences in diet throughout the year. Such extreme changes, as well as the higher variability seen among non-mucoid fecal samples during the mucoid season (**Figure 2**), are often indicative of a dysbiosis between the host and its microbiota (Frank et al., 2007) and a lack of this dysbiosis could explain why the winter season has historically low mucoids.

We also found that the Proteobacteria *Escherichia-Shigella* species (OTU 2) underwent a dramatic increase from winter (0.22 ± 0.12%) to non-mucoid samples (43 ± 8.2%), and the phylum Actinobacteria were absent in winter but present in mucoids. Increases in the abundances of these bacteria are indicative of a dysbiotic event (Darfeuille-Michaud et al., 1998; Baumgart et al., 2007; Krogius-Kurikka et al., 2009; Craven et al., 2012), particularly in humans with inflammatory bowel disease (IBD), a condition that most similarly reflects the chronic mucoids suffered by giant pandas. Therefore, we hypothesize that diet-induced dysbiosis between the giant panda and its gut microbiota, may trigger mucoid episodes similar to dysbiosis-triggering IBD symptoms in humans.

In addition to differences between winter and non-mucoid samples, mucoids were characterized by a number of unique taxa, indicating their divergence from other seasonal changes in the giant panda’s fecal microbiota. Specifically, mucoids contained intermediate abundances of the Firmicutes and Proteobacteria, specifically *Clostridium* (OTU 1), *Turicibacter* (OTU 3), and *Escherichia-Shigella* (OTU 2). This presence indicates that mucoids differ from feces produced in the days surrounding mucoid episodes, and these bacteria may be members of the giant panda GIT microbiota that are shed during these events. Additionally, some differences between the mucoid and both non-mucoid and winter fecal samples were similar to differences between the mucosa- and fecal-associated microbial communities found in other animals (Zoetendal et al., 2002; Malmuthuge et al., 2013). Though not significant, mucoids had a higher abundance of the phyla Bacteroidetes, particularly the class Flavobacteria, and are these taxa are more commonly found associated with the mammalian mucosal lining than with fecal material (Jakobsson et al., 2015). Furthermore, a single unclassified OTU in the family Pasteurellaceae (OTU 11) and a *Streptococcus* (OTU 12) were found only in mucoids, and these taxa are known mucosa-associated bacteria in other animals (Kuhnert and Christensen, 2008; Kaci et al., 2014). Taken together, we speculate that mucoids are a combination of excreted mucosa, along with continued excretion of fecal material following the panda’s switch to leaves.

Although mucoids have been observed throughout the year in captive pandas, they are more frequently observed in the summer months following decreases in dietary fiber (Nickley, 2001; Williams, 2011). A similar relationship has been observed in goats, where decreased fiber intake resulted in changes in the bacterial community and fermentation, leading to a decreased caecal pH and an increased lipopolysaccharide concentration. These alterations to mucosal morphology were associated with intense epithelial damage and local inflammation as a result of dietary change (Metzler-Zebeli et al., 2013; Liu et al., 2014). Given that diet also changed for the giant pandas in our study prior to mucoid collection (**Figure 1**), we speculate that this may also lead to similar mucosal injury, subsequent changes in GIT microbiota, and the need for mucoid excretion.

Under this model, the giant panda’s dramatic shift to the less fibrous leaf portion of bamboo might also cause a change in its mutualistic GIT microbiota. Diet is known to be a major driving force of the GIT microbiota (Flint et al., 2012), and a seasonal shift is evident when comparing winter and non-mucoid samples (**Figures 3** and **4**) both in our study, as well as in previous reports (Williams et al., 2012; Xue et al., 2015). Decreases in dietary fiber and the altered microbiota may then result in inflammation and damage to the mucosal barrier by processes similar to those seen in goats (Metzler-Zebeli et al., 2013; Liu et al., 2014). Without an intact mucosal barrier, the panda experiences further dysbiosis, and fails to maintain its GIT microbiota, as seen by the higher variability within the non-mucoid samples (**Figures 2**–**4**). This dysbiosis, and possibly mucosal damage, reaches a critical level characterized by very low diversity (**Figure 2**). In order to “reset” the system, the mucosal layer along with the altered GIT microbiota is shed, resulting in increased diversity in mucoids, as the mucosa is generally more diverse than feces (Zoetendal et al., 2002; Malmuthuge et al., 2013). Shedding would allow for the reestablishment of the mucosal barrier and a healthier GIT microbiota, as shown by the more winter-like composition and diversity of non-mucoid samples immediately following a mucoid episode (**Figures 2** and **4**). This cycle continues in the weeks following the panda’s sudden change from a culm-rich to a leaf-rich diet until such as time as a stable microbial community and mucosal barrier establish for the new diet. However, this dysbiosis hypothesis remains to be tested in giant pandas.

Interestingly, one mucoid sample from the male panda (day 35) did not fit our proposed model as it had diversity (**Figure 2**) and composition (**Figure 4**) more similar to winter fecal samples. Non-mucoid samples from the same day, both before and after the mucoid episode, had higher diversity and characteristic non-mucoid communities. Importantly, another mucoid with characteristic mucoid diversity and composition occurred the following day (day 36). Thus, we speculate that the day 35 mucoid was a failed shedding event and thus contained more fecal material than mucosa, thereby skewing its observed diversity toward a lower value. Since this shedding failed to remove sufficient mucosa, another episode (day 36) was needed in order to achieve a “reset” of the system.

Giant panda mucoid episodes can have several negative nutritional and health impacts, as giant pandas typically do not feed during these periods. Moreover, these mucoids are more prevalent following the typical breeding season, and reduced nutritional status can be transferred to offspring both during gestation and lactation, potentially impacting cub development. Further work in this area is needed to assess the mucosal injury and dysbiosis hypothesis proposed here. Moreover, investigating possible practices like dietary supplements, might help alleviate GIT distress and the subsequent decline in nutritional status in giant pandas. This work is the first characterization of the mucoid-associated microbiota in giant pandas and serves as an initial step toward elucidating the mechanism behind this phenomenon that affects the overall health of this critically endangered species.

## Author Contributions

CW designed the experiment, and with KD-M, conducted experimentation and data analysis, and wrote the manuscript. MV customized the computing environment that facilitated bioinformatic analyses. AB, GS, DS, SW, and AK aided in data interpretation. AB, GS, DS, and MV provided editorial assistance with the manuscript.

## Conflict of Interest Statement

The authors declare that the research was conducted in the absence of any commercial or financial relationships that could be construed as a potential conflict of interest.

## References

[B1] Baumgart M. Dogan B. Rishniw M. Weitzman G. Bosworth B. Yantiss R. (2007). Culture independent analysis of ileal mucosa reveals a selective increase in invasive *Escherichia coli* of novel phylogeny relative to depletion of Clostridiales in Crohn’s disease involving the ileum. *ISME J.* 1 403–418. 10.1038/ismej.2007.52 18043660

[B2] Bray J. R. Curtis J. T. (1957). An ordination of upland forest communities of southern Wisconsin. *Ecol. Monogr.* 27 325–349. 10.2307/1942268

[B3] Brooks J. P. Edwards D. J. Harwich M. D. Rivera M. C. Fettweis J. M. Serrano M. G. (2015). The truth about metagenomics: quantifying and counteracting bias in 16S rRNA studies. *BMC Microbiol.* 15:66 10.1186/s12866-015-0351-6 PMC4433096 25880246

[B4] Brown A. J. Goldsworthy S. M. Barnes A. A. Eilert M. M. Tcheang L. Daniels D. (2003). The Orphan G protein-coupled receptors GPR41 and GPR43 are activated by propionate and other short chain carboxylic acids. *J. Biol. Chem.* 278 11312–11319. 10.1074/jbc.M211609200 12496283

[B5] Craven M. Egan C. Dowd S. McDonough S. Dogan B. Denkers E. (2012). Inflammation drives dysbiosis and bacterial invasion in murine models of ileal Crohn’s disease. *PLoS ONE* 7:e41594 10.1371/journal.pone.0041594 PMC3404971 22848538

[B6] Darfeuille-Michaud A. Neut C. Barnich N. Lederman E. Martino P. Desreumaux P. (1998). Presence of adherent *Escherichia coli* strains in ileal mucosa of patients with Crohn’s disease. *Gastroenterology* 115 1405–1413. 10.1016/S0016-5085(98)70019-8 9834268

[B7] Davis D. D. (1964). “The giant panda: a morphological study of evolutionary mechanisms,” in *Fieldiana: Zoology Memoirs* Vol. 3 eds Ross L. A. Williams P. M. Nash E. G. (Chicago, IL: Chicago Natural History Museum), 199–218.

[B8] Dierenfeld E. S. Hintz H. F. Robertson J. B. Van Soest P. J. Oftedal O. T. (1982). Utilization of bamboo by the giant panda. *J. Nutr.* 112 636–641. 6279804 10.1093/jn/112.4.636

[B9] Edwards M. S. Zhang G. Wei R. Liu X. (2006). “Nutrition and dietary husbandry,” in *Giant Pandas: Biology, Veterinary Medicine and Management*, eds Wildt D. E. Zhang A. Zhang H. Janssen D. L. Ellis S. (Cambridge: Cambridge University Press), 101–158.

[B10] Fava F. Danese S. (2011). Intestinal microbiota in inflammatory bowel disease: friend of foe? *World J. Gastroenterol.* 5 557–566. 10.3748/wjg.v17.i5.557 21350704 PMC3040327

[B11] Flint H. Scott K. Louis P. Duncan S. (2012). The role of the gut microbiota in nutrition and health. *Nat. Rev. Gastroenterol. Hepatol.* 9 577–589. 10.1038/nrgastro.2012.156 22945443

[B12] Frank D. N. St Amand A. L. Feldman R. A. Boedeker E. C. Harpaz N. Pace N. R. (2007). Molecular-phylogenetic characterization of microbial community imbalances in human inflammatory bowel diseases. *Proc. Natl. Acad. Sci. U.S.A.* 104 13780–13785. 10.1073/pnas.0706625104 17699621 PMC1959459

[B13] Good I. J. (1953). The population frequencies of species and the estimation of population parameters. *Biometrika* 40 237–264. 10.1093/biomet/40.3-4.237

[B14] Hansen R. L. Carr M. M. Apanavicius C. J. Jiang P. Bissel H. A. Gocinski B. L. (2010). Seasonal shifts in giant panda feeding behavior: relationships to bamboo plant part consumption. *Zoo Biol.* 29 470–483. 10.1002/zoo.20280 19862794

[B15] Hooper L. Midtvedt T. Gordon J. (2002). How host-microbial interactions shape the nutrient environment of the mammalian intestine. *Annu. Rev. Nutr.* 22 283–307. 10.1146/annurev.nutr.22.011602.092259 12055347

[B16] Illumina (2013). *16S Metagenomic Sequencing Library Preparation.* Available at: www.illumina.com (accessed October 27, 2014).

[B17] Ito K. Sakai S. (2009). Optimal defense strategy against herbivory in plants: conditions selecting for induced defense, constitutive defense, and no-defense. *J. Theor. Biol.* 260 453–459. 10.1016/j.jtbi.2009.07.002 19591847

[B18] Jaccard P. (1912). The distribution of the flora in the alpine zone. *New Phytol.* 11 37–50. 10.1111/j.1469-8137.1912.tb05611.x

[B19] Jakobsson H. E. Rodríguez-Piñeiro A. M. Schütte A. Ermund A. Boysen P. Bemark M. (2015). The composition of the gut microbiota shapes the colon mucus barrier. *EMBO Rep.* 16 164–177. 10.15252/embr.201439263 25525071 PMC4328744

[B20] Janssen D. L. Edwards M. S. Sutherland-Smith M. Yu J. Q. Li D. S. Zhang G. Q. (2006). “Significant medical issues and biological reference values for giant pandas from the biomedical survey,” in *Giant Pandas: Biology, Veterinary Medicine and Management*, eds Wildt D. E. Zhang A. Zhang H. Janssen D. L. Ellis S. (Cambridge: Cambridge University Press), 59–85.

[B21] Jin C. Ciochon R. L. Dong W. Hunt R. M. Liu J. Jaeger M. (2007). The first skull of the earliest giant panda. *Proc. Natl. Acad. Sci. U.S.A.* 104 10932–10937. 10.1073/pnas.0704198104 17578912 PMC1904166

[B22] Johansson M. E. Larsson J. M. Hansson G. C. (2011). The two mucus layers of colon are organized by the MUC2 mucin, whereas the outer layer is a legislator of host–microbial interactions. *Proc. Natl. Acad. Sci. U.S.A.* 108 4659–4665. 10.1073/pnas.1006451107 20615996 PMC3063600

[B23] Kaci G. Goudercourt D. Dennin V. Pot B. Doré J. Ehrlich D. S. (2014). Anti-inflammatory properties of *Streptococcus salivarius*, a commensal bacterium of the oral cavity and digestive tract. *Appl. Environ. Microbiol.* 80 928–934. 10.1128/AEM.03133-13 24271166 PMC3911234

[B24] Klindworth A. Pruesse E. Schweer T. Peplies J. Quast C. Horn M. (2013). Evaluation of general 16S ribosomal RNA gene PCR primers for classical and next-generation sequencing-based diversity studies. *Nucleic Acids Res.* 41:e1 10.1093/nar/gks808 PMC3592464 22933715

[B25] Kozich J. J. Westcott S. L. Baxter N. T. Highlander S. K. Schloss P. D. (2013). Development of a dual-index sequencing strategy and curation pipeline for analyzing amplicon sequence data on the MiSeq Illumina sequencing platform. *Appl. Environ. Microbiol.* 79 5112–5120. 10.1128/AEM.01043-13 23793624 PMC3753973

[B26] Krogius-Kurikka L. Lyra A. Malinen E. Aarnikunnas J. Tuimala J. Paulin L. (2009). Microbial community analysis reveals high level phylogenetic alterations in the overall gastrointestinal microbiota of diarrhoea-predominant irritable bowel syndrome sufferers. *BMC Gastroenterol.* 9:95 10.1186/1471-230X-9-95 PMC2807867 20015409

[B27] Kuhnert P. Christensen H. (2008). *Pasteurellaceae: Biology, Genomics and Molecular Aspects.* Norfolk, VA: Caister Academic Press.

[B28] Liu J. Xu T. Zhu W. Mao S. (2014). High-grain feeding alters caecal bacterial microbiota composition and fermentation and results in caecal mucosal injury in goats. *Br. J. Nutr.* 112 416–427. 10.1017/S0007114514000993 24846282

[B29] Loeﬄer I. K. Montali R. J. Rideout B. A. (2006). “Disease and pathology of giant pandas,” in *Giant Pandas: Biology, Veterinary Medicine and Management*, eds Wildt D. E. Zhang A. Zhang H. Janssen D. L. Ellis S. (Cambridge: Cambridge University Press), 101–158.

[B30] Louis P. Flint H. (2009). Diversity, metabolism and microbial ecology of butyrate-producing bacteria from the human large intestine. *FEMS Microbiol. Lett.* 294 1–8. 10.1111/j.1574-6968.2009.01514.x 19222573

[B31] Malmuthuge N. Li M. Goonewardene L. A. Oba M. Guan L. L. (2013). Effect of calf starter feeding on gut microbial diversity and expression of genes involved in host immune responses and tight junctions in dairy calves during weaning transition. *J. Dairy Sci.* 96 3189–3200. 10.3168/jds.2012-6200 23498024

[B32] Metzler-Zebeli B. Schmitz-Esser S. Klevenhusen F. Podstatzky-Lichtenstein L. Wagner M. Zebeli Q. (2013). Grain-rich diets differently alter ruminal and colonic abundance of microbial populations and lipopolysaccharide in goats. *Anaerobe* 20 65–73. 10.1016/j.anaerobe.2013.02.005 23474085

[B33] Nickley J. K. (2001). *Nutrient Composition of Bamboos Fed to Captive Giant Pandas (Ailuropoda melanoleuca) and the Relationship between Bamboo Intake and Fecal Consistency.* Pomona, CA: California Polytechnical University-Pomona.

[B34] Oksanen J. Blanchet F. G. Kindt R. Legendere P. Minchin P. R. O’Hara R. B. (2015). *Vegan: Community Ecology Package. R Package Vegan, Version 2.2-1.* Available at: http://cran.r-project.org

[B35] Pruesse E. Quast C. Knittel K. Fuchs B. Ludwig W. Peplies J. (2007). SILVA: a comprehensive online resource for quality checked and aligned ribosomal RNA sequence data compatible with ARB. *Nucleic Acids Res.* 35 7188–7196. 10.1093/nar/gkm864 17947321 PMC2175337

[B36] Qiu X. M. Mainka S. A. (1993). Review of mortality of the giant panda (*Ailuropoda melanoleuca*). *J. Zoo Wildl. Med.* 24 425–429.

[B37] R Core Team (2015). *R: A Language and Environment for Statistical Computing.* Vienna: R Foundation for Statistical Computing Available at: http://www.R-project.org/

[B38] Schaller G. B. Hu J. Pan W. Zhu J. (1985). *The Giant Pandas of Wolong.* Chicago, IL: University of Chicago Press.

[B39] Schloss P. D. Handelsman J. (2005). Introducing DOTUR, a computer program for defining operational taxonomic units and estimating species richness. *J. Appl. Environ. Microbiol.* 71 1501–1506. 10.1128/AEM.71.3.1501-1506.2005 PMC1065144 15746353

[B40] Shepard R. N. (1966). Metric structures in ordinal data. *J. Math. Psychol.* 3 287–315. 10.1016/0022-2496(66)90017-4

[B41] Steinman K. J. Monfort S. T. McGeehan L. Kersey D. C. Gual-Sil F. Snyder R. J. (2006). “Endocrinology of the giant panda and application of hormone technology to species management,” in *Giant Pandas: Biology, Veterinary Medicine and Management*, eds Wildt D. E. Zhang A. Zhang H. Janssen D. L. Ellis S. (Cambridge: Cambridge University Press), 101–158.

[B42] Stevenson D. M. Weimer P. J. (2007). Dominance of *Prevotella* and low abundance of classical ruminal bacterial species in the bovine rumen revealed by relative quantification real-time PCR. *Appl. Microbiol. Biotechnol.* 75 165–174. 10.1007/s00253-006-0802-y 17235560

[B43] Tarou L. R. Williams J. Powell D. M. Tabet R. Allen M. (2005). Behavioral preferences for bamboo in a pair of captive giant pandas (*Ailuropoda melanoleuca*). *Zoo Biol.* 24 177–183. 10.1002/zoo.20038

[B44] Wagner Mackenzie B. Waite D. W. Taylor M. W. (2015). Evaluating variation in human gut microbiota profiles due to DNA extraction method and inter-subject differences. *Front. Microbiol.* 6:130 10.3389/fmicb.2015.00130 PMC4332372 25741335

[B45] Williams C. L. (2011). *The Effect of Dietary Changes on Microbial Populations within the Gastrointestinal Tract of the Giant Panda (Ailuropoda melanoleuca) [Electronic Resource].* Mississippi State, MS: Mississippi State University.

[B46] Williams C. L. Willard S. Kouba A. Sparks D. Holmes W. Falcone J. (2012). Dietary shifts affect the gastrointestinal microflora of the giant panda (*Ailuropoda melanoleuca*). *J. Anim. Physiol. Anim. Nutr.* 97 577–585. 10.1111/j.1439-0396.2012.01299.x 22524500

[B47] Xue Z. Zhang W. Wang L. Hou R. Zhang M. Fei L. (2015). The bamboo-eating giant panda harbors a carnivore-like gut microbiota, with excessive seasonal variations. *Mbio* 6:e00022-15 10.1128/mBio.00022-15 PMC4442137 25991678

[B48] Zhang Z. H. Zhang A. J. Hou R. Wang J. S. Li G. H. Fei L. S. (2006). “Historical perspective of breeding giant pandas ex situ in china an high priorities for the future,” in *Giant Pandas: Biology, Veterinary Medicine and Management*, eds Wildt D. E. Zhang A. Zhang H. Janssen D. L. Ellis S. (Cambridge: Cambridge University Press), 101–158.

[B49] Zhu L. Wu Q. Dai J. Zhang S. Wei F. (2011). Evidence of cellulose metabolism by the giant panda gut microbiome. *Proc. Natl. Acad. Sci. U.S.A.* 108 17714–17719. 10.1073/pnas.1017956108 22006317 PMC3203778

[B50] Zoetendal E. Wright A. Vilpponen-Salmela T. Ben-Amor K. Akkermans A. Vos W. (2002). Mucosa-associated bacteria in the human gastrointestinal tract are uniformly distributed along the colon and differ from the community recovered from feces. *Appl. Environ. Microbiol.* 68 3401–3407. 10.1128/AEM.68.7.3401-3407.2002 12089021 PMC126800

