# Supplementary material for: Dietary Shifts May Trigger Dysbiosis and Mucous Stools in Giant Pandas (Ailuropoda melanoleuca)
Source: Front Microbiol. 2016 May 6;7:661. doi: 10.3389/fmicb.2016.00661 (PMC4858621; doi:10.3389/fmicb.2016.00661)
Supplement: Supplementary file 1 [file Data_Sheet_1.PDF]

Supplementary Material

Article Title

Candace L Williams, Kimberly A. Dill-McFarland, Michael W. Vandewege, Darrell L. Sparks, Scott T. Willard, Andrew J. Kouba, Garret Suen*, Ashli E. Brown*

*** Correspondence:** Garret Suen: gsuen@wisc.edu; Ashli Brown: abrown@mscl.msstate.edu

1 Supplementary Figures and Tables

1.1 Supplementary Figures

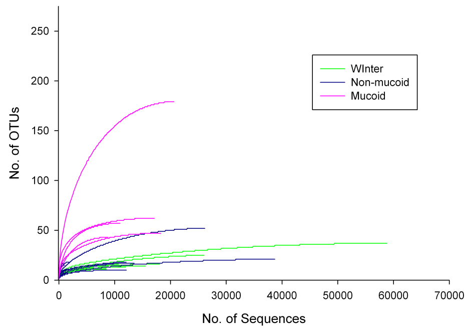

Supplementary Figure 1. Rarefaction curves for all winter, non-mucoid, and mucoid samples analyzed.

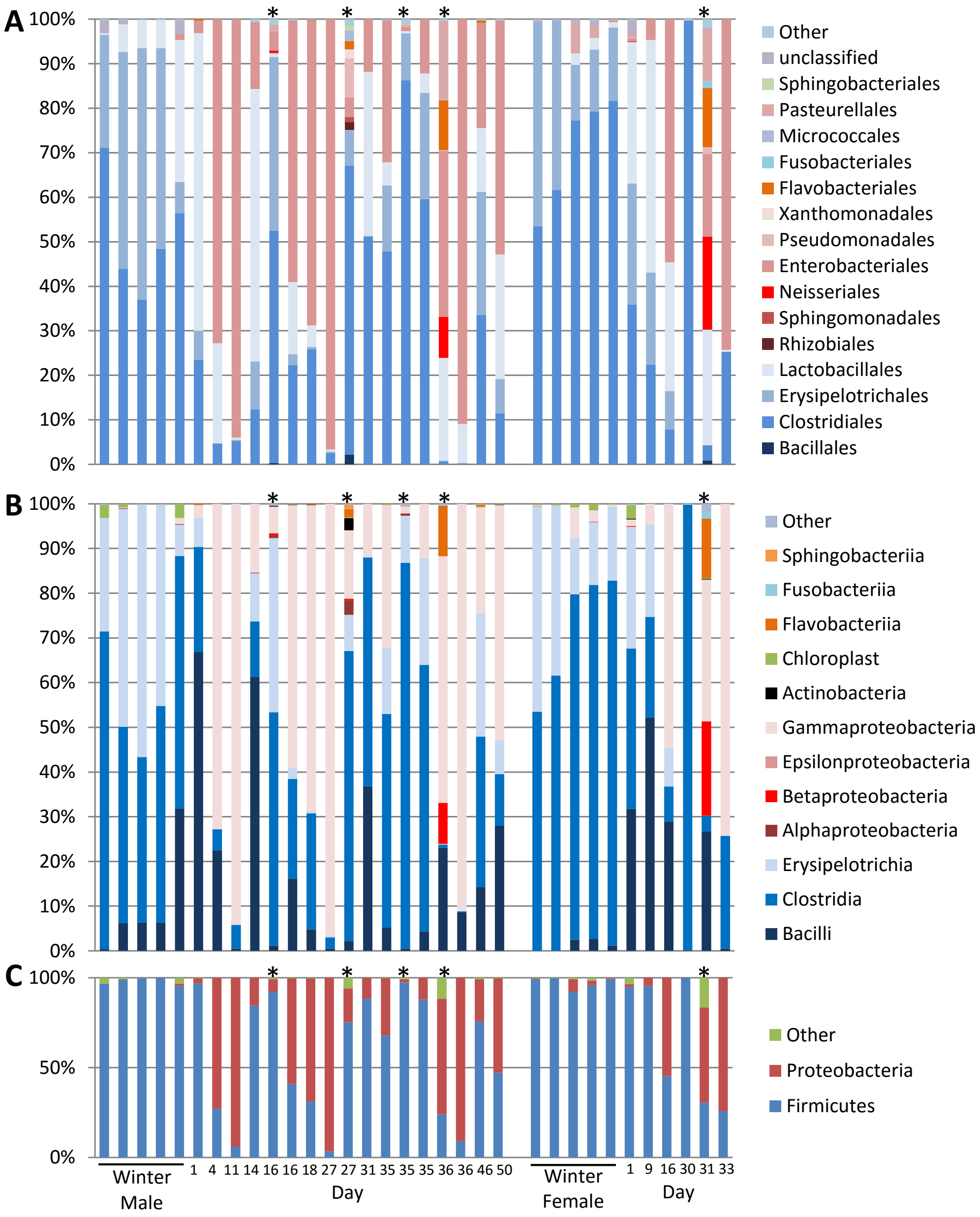

Figure S2. Relative abundances of taxa at the A) order, B) class, and C) phyla levels. Only taxa at > 1% relative abundance in at least one sample are shown. All classes and orders within the Firmicutes are grouped together in shades of blue, and all within the Proteobacteria are shades of red. Mucoids are indicated by (*).

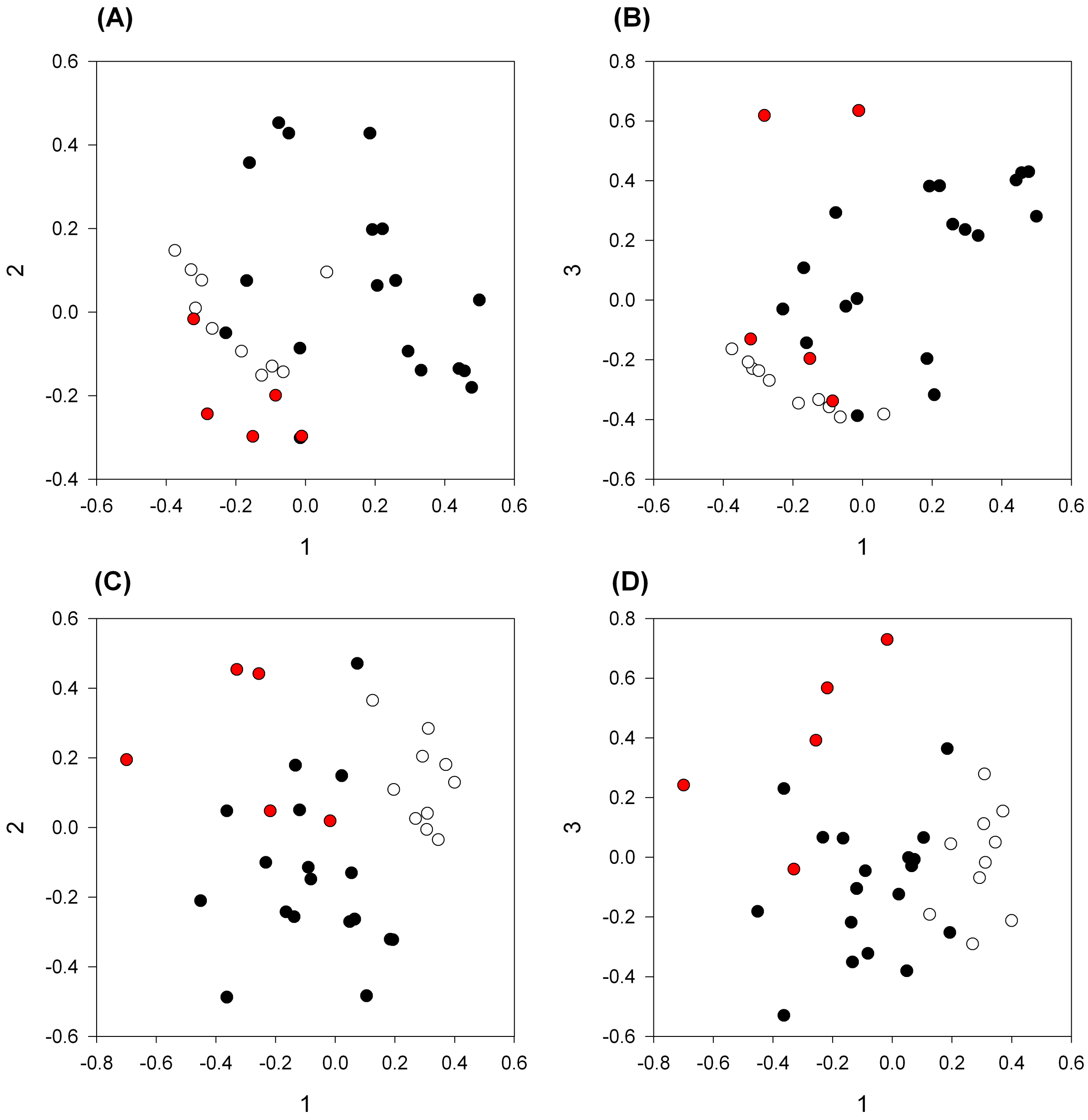

Figure S3. Two-dimensional nonmetric multidimensional scaling analysis showing differences in (A,B) community structure (Bray-Curtis, lowest stress: 0.0810, R-square: 0.965) and (C,D) community composition (Jaccard, lowest stress: 0.192, R-square: 0.766) of winter, non-mucoid, and mucoid samples in giant pandas.

1.2 Supplementary Tables

Supplementary Table 1. Sample IDs, Collection Date, and Sample Type for samples in the dataset. a-c in the collection date indicates the order in which samples occurred on a given date

Sample ID	Collection date	Sample type
**Male**		
M1	2/12/13	Winter
M2	2/12/13	Winter
M3	2/12/13	Winter
M4	2/12/13	Winter
M5	2/12/13	Winter
N1	7/4/14	Non-mucoid
N2	7/7/14	Non-mucoid
N3	7/14/14	Non-mucoid
N4*	7/17/14 [a]	Mucoid
N5	7/17/14 [b]	Non-mucoid
M6	7/19/14[a]	Mucoid
N7	7/19/14[b]	Non-mucoid
N8	7/21/14	Non-mucoid
N10	7/30/14 [a]	Non-mucoid
M11	7/30/14 [b]	Mucoid
N12	8/3/14	Non-mucoid
N13	8/7/14 [a]	Non-mucoid
M14	8/7/14 [b]	Mucoid
N15	8/7/14 [c]	Non-mucoid
M16	8/8/14 [a]	Mucoid
N17	8/8/14 [b]	Non-mucoid
N18	8/18/14	Non-mucoid
N20	8/22/14	Non-mucoid
**Female**		
F1	2/12/13	Winter
F2	2/12/13	Winter
F3	2/12/13	Winter
F4	2/12/13	Winter
F5	2/12/13	Winter
N21	6/29/14	Non-mucoid
N22	7/7/14	Non-mucoid
N23	7/14/14	Non-mucoid
N25	7/28/14	Non-mucoid
M26	7/29/14	Mucoid
N27	7/31/14	Non-mucoid

*Failed to sequence

Supplementary Table 2. Number of sequences, estimated coverage, diversity and OTU richness in giant panda winter, non-mucoid, and mucoid samples.

Sample	16S rRNA reads		Community Richness		Community Diversity		
	Total	High-quality	Inverse Berger-Parker	Shannon	Inverse-Simpson	Number of OTUs	Good's coverage
**Winter** (n=10)	214,655	171,373	1.7 ± 0.11	0.96 ± 0.040	2.1 ± 0.10	14	>0.99
**Non-mucoid** (n=18)	151,401	128,239	1.9 ± 0.20	1.1 ± 0.13	2.6 ± 0.32	43	>0.99
**Mucoid** (n=5)	91,302	75,794	2.6 ± 0.57	1.7 ± 0.26	4.0 ± 1.0	84	>0.99

Mean values and standard error given.

Supplementary Table 3. *P*-values of animal and randomized control differences within individual sample types (Winter and Non-mucoid) with respect to community structure and composition at the phyla, family, and OTU level.

	**Phyla**		**Family**		**OTU**	
	Animal	Control	Animal	Control	Animal	Control
**Winter**						
Bray-Curtis	0.53	0.43	0.11	0.71	0.11	0.71
Jaccard	0.53	0.43	0.11	0.71	0.11	0.72
**Non-mucoid**						
Bray-Curtis	0.40	0.51	0.31	0.36	0.33	0.25
Jaccard	0.40	0.50	0.30	0.36	0.32	0.25

Supplementary Table 4. *P*-values of sample type and randomized control differences with respect to community structure and composition at the phyla, family, and OTU level.

	**Phyla**		**Family**		**OTU**	
	Sample	Control	Sample	Control	Sample	Control
**Bray-Curtis**	0.035*	0.93	0.00030*	0.98	0.00040*	0.99
**Jaccard**	0.040*	0.93	0.00070*	0.98	0.00040*	0.99

* Significant.

Supplementary Table 5. *P*-values of sample type, animal, and randomized control differences within combined sample types (Winter and Non-mucoid) with respect to community structure and composition at the phyla, family, and OTU level.

	**Phyla**			**Family**			**OTU**		
	Sample	Animal	Control	Sample	Animal	Control	Sample	Animal	Control
**Bray-Curtis**	0.035*	0.74	0.74	0.00040*	0.24	0.72	0.00080*	0.28	0.60
**Jaccard**	0.034*	0.73	0.73	0.00060*	0.23	0.71	0.00050*	0.28	0.61
